# Improved Oxidation of Naringenin to Carthamidin and Isocarthamidin by *Rhodotorula marina*

**DOI:** 10.1007/s12010-014-0787-4

**Published:** 2014-03-11

**Authors:** Anna Madej, Jarosław Popłoński, Ewa Huszcza

**Affiliations:** Department of Chemistry, Wrocław University of Environmental and Life Sciences, Norwida 25, 50-375 Wrocław, Poland

**Keywords:** Naringenin, Carthamidin, Isocarthamidin, Biotransformation *Rhodotorula marina*

## Abstract

A novel single-step microbial transformation process for the efficient production of carthamidin and isocarthamidin from naringenin by yeast *Rhodotorula marina* in an aerated bioreactor was described. The biotransformation led to the total product concentration of 233 mg/l. The highest conversion efficiency observed for carthamidin was 0.31 mg/mg of naringenin and for isocarthamidin 0.47 mg/mg of naringenin.

## Introduction

Flavonoids are polyphenolic compounds of plant origin that are important constituents of the human diet. They have been reported to possess a wide spectrum of health-promoting properties, including antibacterial, antiviral, estrogenic, anti-obesity, and anticancer activities, which are useful in the treatment of several human pathologies [1].

**Fig. 1:**
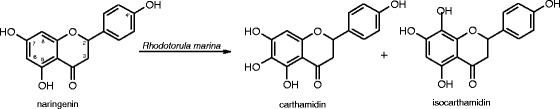
Transformation of naringenin by *Rhodotorula marina*

Naringenin (Fig. 1) is one of the most abundant flavonoids found in grapes, tomatoes, and citrus fruits. This flavanone has gained increasing interest in recent years because of its wide range of biological activity, including antioxidant [2], anti-inflammatory [3], antimicrobial [4, 5], antiviral [6], antimutagenic [7], antiestrogenic [8], antiatherogenic [9], hepatoprotective [10, 11], nephroprotective [12], and anticancer [13, 14] action. Positive effects of naringenin on lipid metabolism [15, 16] and plasma glucose levels [16–19] have also been reported.

Since naringenin is a compound isolated from the agro-residue, it is relatively inexpensive. Not only does it serve as an excellent and frequently employed model flavonoid but also it is a leading substance for the preparation of new biologically active derivatives. For this purpose both chemical and biotransformation methods have been used. Chemical modifications involved acylation [20] and *O*-alkylation [20, 21] while biotransformations using microorganisms, plant cultured cells, and pure enzymes led to glycosylation [22], *O*-alkylation [23], acylation [24], halogenation [25], sulfation [26, 27], C–C coupling [28], and hydroxylation [29–32].

The last of these modifications, conducted by the fungi of the genus *Aspergillus*, afforded carthamidin (6-hydroxynaringenin) and isocarthamidin (8-hydroxynaringenin). These metabolites exhibited much stronger antioxidant activity than both naringenin and its glycoside naringin [29]. The activity of the isocarthamidin is comparable to that of α-tocopherol, whereas carthamidin is slightly less active. Kim et al. [33] suggested that these compounds obtained by fermentation of *Dangyuja* (Citrus grandis Osbeck) extract increased also the antidiabetic activity of the raw material used. A novel antidiabetic agent which contains carthamidine and/or isocarthamidin as active ingredients was also patented [34].

Recently, 8-hydroxynaringenin has been identified as a suicide substrate of mushroom tyrosinase [35], which greatly expands the range of its possible applications. Tyrosinase is a monooxygenase known to be the key enzyme in melanin biosynthesis, by catalyzing the oxidation of phenol to *O*-chinone, observed in the early stage of various browning phenomena in nature [36, 37]. Tyrosinase inhibitors can therefore be useful for the control of enzymatic browning during fruit and vegetable pulp manufacturing [38], for the treatment of some dermatological disorders associated with melanin hyperpigmentation [39] and neurodegeneration [40, 41], for whitening and depigmentation in cosmetics [42, 43], and for insect control [44].

For the above reasons and because of low content of carthamidin and isocarthamidin in plant material [45–48], it is desirable to develop effective methods of obtaining carthamidin and isocarthamidin from naringenin. Biotransformation is a method that allows for regioselective hydroxylation of the substrate and, at the same time, preserves the status of the products as “natural compounds.” Therefore, it is particularly useful in this respect.

This paper describes biotransformation leading to carthamidin and isocarthamidin, carried out on a preparative scale and using yeast as a biocatalyst.

## Materials and Methods

### Microorganism

The yeast strain *Rhodotorula marina* AM77 was purchased from Institute of Biology and Botany of the Wrocław Medical University, Poland.

### Substrate

Naringenin was purchased from Sigma-Aldrich.

### Products

Carthamidin (5, 6, 7, 4′- tetrahydroxyflavanone) ^1^H NMR (600 MHz, DMSO-*d*_6_) δ (ppm): 2.65 (dd, 1H, *J* = 17.1; 3.0 Hz, H-3 eq); 3.22 (dd, 1H, *J* = 17.1; 12.3 Hz, H-3ax); 5.36 (dd, 1H, *J* = 12.8; 2.9 Hz, H-2); 5.94 (s, 1H, H-8); 6.79 (d, 2H, *J* = 8.5 Hz, H-3′, 5′); 7.31 (d, 2H, *J* = 8.6 Hz, H-2′,6′); 8.11 (s, 1H, 7–OH); 9.57 (s, 1H, 4′–OH); 10.42 (s, 1H, 6-OH); 11.98 (s, 1H, 5-OH). ^13^C NMR (DMSO-*d*_6_) δ (ppm): 42.32 (C-3); 78.50 (C-2); 94.73 (C-8); 101.71 (C-10); 115.16 (C-3′, 5′); 126.32 (C-6); 128.30 (C-2′, 6′); 129.19 (C-1′); 150.19 (C-9); 155.27 (C-5); 155.77 (C-7); 157.65 (C-4′); 196.66 (C-4). HR ESI-MS *m*/*z*: 287.0520 [M–H]^−^ (calcd for C_15_H_12_O_6_–H, 287.0556); UV (MeOH) λ_max_ 292.7 nm.

Isocarthamidim (5, 7, 8, 4′- tetrahydroxyflavanone) ^1^H NMR (600 MHz, DMSO-*d*_6_) δ (ppm): 2.71 (dd, 1H, *J* = 17.1; 3.1 Hz, H-3 eq); 3.22 (dd, 1H, *J* = 17.1; 12.3 Hz, H-3ax); 5.42 (dd, 1H, *J* = 12.3; 2.9 Hz, H-2); 5.93 (s, 1H, H-6); 6.79 (d, 2H, *J* = 8.5 Hz, H-3′, 5′); 7.35 (d, 2H, *J* = 8.5 Hz, H-2′, 6′); 8.11 (s, 1H, 7-OH); 9.57 (s, 1H, 4′-OH); 10.42 (s, 1H, 8-OH); 11.75 (s, 1H, 5-OH). ^13^C NMR (DMSO-*d*_6_) δ (ppm): 42.28 (C-3); 78.50 (C-2); 95.44 (C-6); 101.71 (C-10); 115.12 (C-3′,5′); 125.61 (C-8); 128.40 (C-2′, 6′); 129.19 (C-1′); 149.41 (C-9); 156.51 (C-5); 155.81 (C-7); 157.65 (C-4′); 196.62 (C-4). HR ESI-MS *m*/*z*: 287.05200 [M-H]^−^ (calcd for C_15_H_12_O_6_–H, 287.0556); UV (MeOH) λ_max_ 293.9 nm.

### Biotransformation Procedure

The growth medium for inoculum preparation and for biotransformation process (Sabouraud medium) contained 3 % glucose and 1 % bacto peptone (Difco). A seed culture was grown in a six 300-ml flasks (each containing 50 ml of the growth medium) on a shaker at 28 °C for 2 days to obtain the cell dry weight of *ca*. 7 g/L. The inoculum in amount of 300 ml was introduced into a 5-l fermenter (Biostat B Plus, Sartorius, Germany), containing 1.7 l of the medium. After 24 h of cultivation at 28 °C, 600 mg of naringenin dissolved in 15 ml of methanol was added. The aeration rate was fixed at 0.6 *v*/*v*/*m*. The stirrer speed was adjusted to 600 rpm, and the dissolved oxygen concentration was maintained at 45 ± 5 % saturation. The progress of naringenin conversion was monitored by thin layer chromatography (TLC) and high-performance liquid chromatography (HPLC). This procedure was repeated three times. Substrate control sample consisted of naringenin and a sterile growth medium incubated without a microorganism.

### Sample Preparation

Five milliliters of the culture medium was acidified with 1 M HCl to pH 4 and extracted three times with 7.5 ml of ethyl acetate. The combined extracts were evaporated and the residue dissolved in methanol and analyzed by TLC and HPLC.

### Products Isolation and Analysis

TLC was carried out on Merck silica gel 60 F_254_ (0.2 mm thick) plates. Compounds were detected by spraying the plates with 1 % Ce(SO_4_)_2_ and 2 % H_3_[P(Mo_3_O_10_)_4_] in 10 % H_2_SO_4_. HPLC was performed on a Waters 2695 Aliance instrument with a photodiode array detector Waters 2996 (detection at 290-nm wavelength) using the analytical HPLC column Agilent ZORBAX Eclipse XDB 5 μm (46 × 250 mm). Separation conditions: flow rate of 0.8 ml/min; mobile phase A: acetonitrile; mobile phase B: 0.1 % acetic acid in water. Chromatographic separation was achieved using the gradient elution: linear gradient of A from 25 to 38 % over 10 min, isocratic elution of A 38 % for 18 min, linear gradient of A from 38 to 25 % over 2 min, and isocratic elution of A 25 % for 6 min.

When the biotransformation was complete, the culture was acidified with 1 M HCl to pH 4 and extracted three times with 0.5 L of ethyl acetate. The combined organic phases were evaporated and the products separated by column chromatography on Sephadex LH-20 gel (Pharmacia) using chloroform/methanol (8:2 *v*/*v*) as eluent. Quantitative analysis of the ethyl acetate extract of the biotransformation mixture was performed by means of HPLC, using calibration curves for the substrate and for all the isolated products.

Nuclear magnetic resonance (NMR) spectra (^1^H NMR, ^13^C NMR, DEPT 135°, ^1^H–^1^H NMR (COSY), and ^1^H–^13^C NMR (HMQC)) were recorded on a MHz DRX Bruker Avance™ 600 (600 MHz) instrument in DMSO-*d*_6_ (Sigma-Aldrich). UV spectra were run on a Spectrofotometer Cintra 303, GBC, in methanol (Merck). Negative-ion electrospray ionization tandem mass spectrometry (ESI-MS) spectra were measured on a Bruker micrOTOF-Q spectrometer.

### Free Radical-Scavenging Activity Assay

Free radical-scavenging activity was measured essentially by the method proposed by Sharma and Bhat [49] using 1,1-diphenyl-2-picrylhydrazyl (DPPH, Sigma-Aldrich). Briefly, 1 ml of 0.5 mM methanolic solution of DPPH radical was added to 2 ml of methanol and 2 ml of methanolic test samples of different concentrations. Final concentration of DPPH in each sample was 100 μM (0.5 μmol). The solvents and other chemicals were of analytical grade. The mixtures were shaken vigorously and left to stand for 30 min at room temperature in the dark. The absorbance at 517 nm by DPPH was measured by UV–vis spectrophotometry. Naringenin was used as a control.

The percentage of inhibition of the DPPH radical by the samples was calculated as follows:

$$ \mathrm{Inhibition}\left[\%\right]=100\times \left({{\mathrm{ABS}}_t}_{=0}-{{\mathrm{ABS}}_t}_{=30}\right)/{{\mathrm{ABS}}_t}_{=0}, $$

where ABS_*t*=0_ was the absorbance of DPPH at time 0 and ABS_*t*=30_ was the absorbance of DPPH after 30 min of incubation. Sample concentration providing 50 % of inhibition (IC_50_) was calculated from the graph plotting inhibition percentage against compound concentration in a sample. All measurements were made in triplicates. One-way analysis of variance (ANOVA) was performed with SPSS. The differences were considered significant at *p* < 0.05 using the post hoc evaluation with Fisher’s least significant difference test.

## Results and Discussion

Recently, we reported the ability of the yeast *Rhodoorula marina* to transform naringenin into polyhydroxylated products: carthamidin and isocarthamidin [31]. In those studies, the biotransformation was carried out in Erlenmeyer flasks, with the maximum initial concentration of naringenin of 300 mg/l. The present research focuses on performing this reaction in a larger scale, using a bioreactor.

The products of naringenin biotransformation—carthamidin and isocarthamidin—were unambiguously identified by spectroscopic methods (NMR, UV, and HR ESI-MS).

**Fig. 2:**
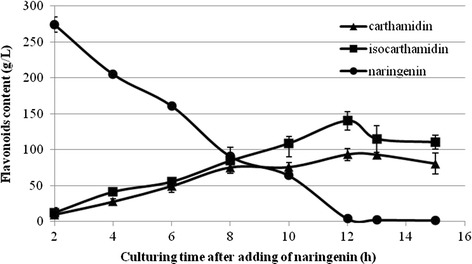
The course of naringenin transformation in the *Rhodotorula marina* culture (determined by HPLC)

**Fig. 3:**
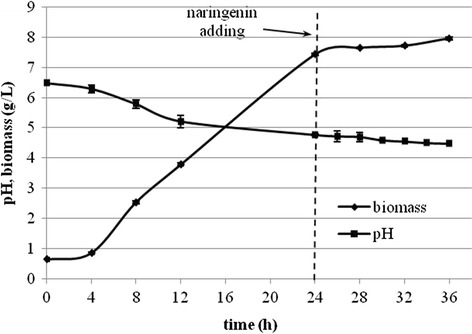
Growth curve of *Rhodotorula marina* on 2-l scale in a fermenter

Figure 2 shows the time course of bioconversion of naringenin run in a 2.0-l batch culture. To a 24-h-old culture of the dry biomass concentration of 7.4 g/l (Fig. 3), 600 mg of the substrate (300 mg/l) was added. Both products appeared simultaneously and accumulated in the reaction mixture at highest rate over 12 h.

**Fig. 4:**
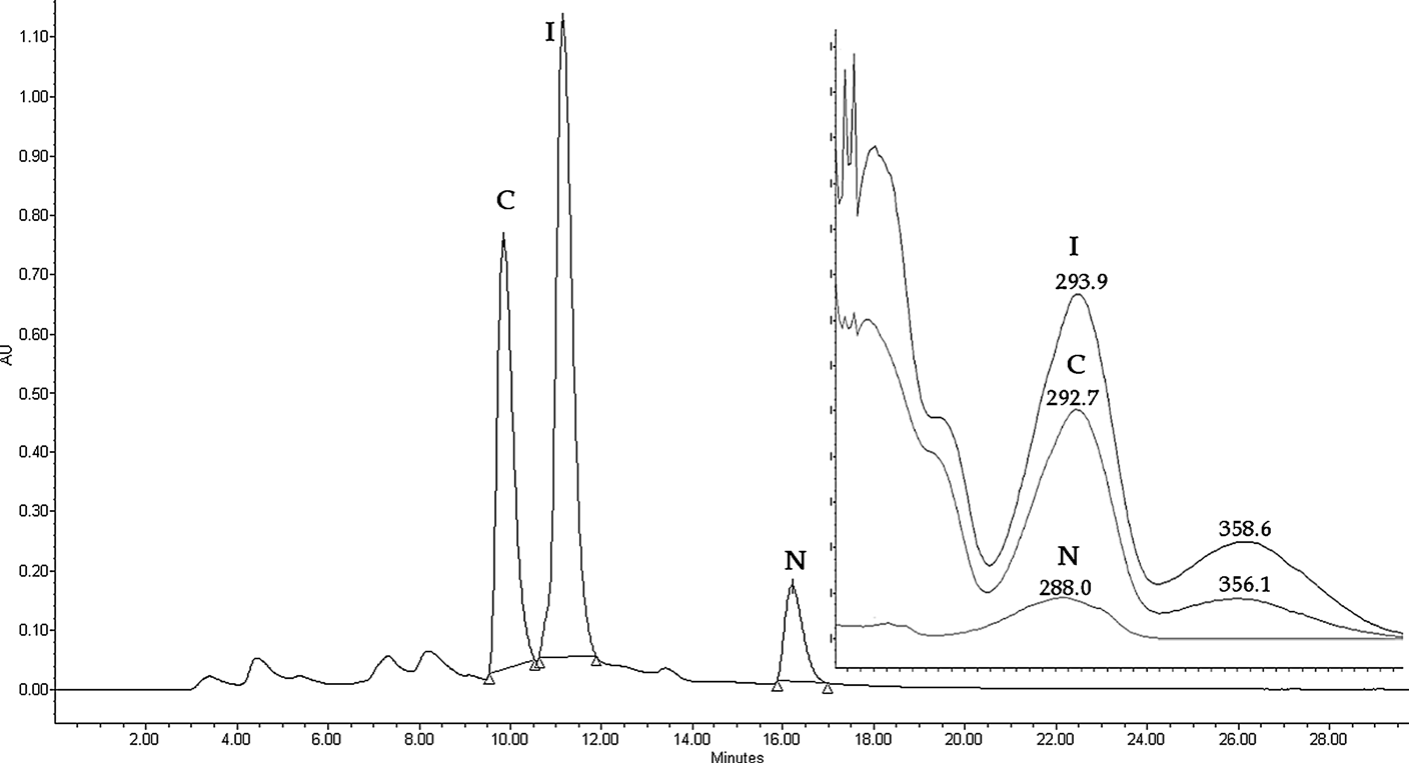
HPLC elution profile of extract of 12-h biotransformation mixture; *N* naringenin, *C* carthamidin, *I* isocarthamidin

Maximum conversion efficiency of carthamidin was 0.31 mg/mg of naringenin and that of isocarthamidin was 0.47 mg/mg of naringenin. At this stage of the biotransformation, the total concentration of the flavonoids in the bioreactor determined by HPLC (Fig. 4) was found to be 237.3 mg/l (93.3 mg/l for carthamidin, 140.0 mg/l for isocarthamidin, and 4.0 mg/l for naringenin). The specific production rate for the mixture of carthamidin and isocarthamidin reached the value of 0.065 g/gl and the maximal yield of 0.399 g/g. Both microbial growth time and biotransformation time were considerably shorter compared to the cultures cultivated in shaken Erlenmeyer flasks. In a 24-h-old culture, we obtained the cell dry biomass of 7.43 g/L, whereas such a value was observed earlier for a 41-h-old culture [31]. Biotransformation time was also much shorter, as after 12 h in the bioreactor, there was no more naringenin observed, while in Erlenmeyer flasks, it was present in amount of 83 % of the flavonoid mixture.

In the laboratory scale studies conducted by Xu et al. (2012) using *Aspergillus niger* as a biocatalyst, similar conversion of the substrate to both products was observed but at significantly lower concentrations. The maximum concentration of isocarthamidin was 21.7 mg/l and carthamidin 21.5 mg/l, with the initial concentration naringenin equal to 60 mg/l. Another major advantage of our method is the short reaction time.

The optimal time of cultivation and biotransformation using *A. niger* was 3–4 days, whereas a 24-h-old culture of *R. marina* performed the reaction in 12 h.

Moreover, biotransformations by yeasts are generally simpler and more convenient compared to molds.

The antioxidant activity was checked for the mixture of 12-h biotransformation extract and for the mixture of carthamidin and isocarthamidin (1:8) obtained after the purification of this extract. The 2,2′-diphenyl-1-picrylhydrazyl (DPPH) radical scavenging method was applied, using commercial naringenin as a control. The IC_50_ values were found as follows: 14.3 mg/l for the 12-h biotransformation mixture extract, 4.0 mg/l for purified hydroxylated products and 50.1 mg/l for naringenin. It was demonstrated that the mixture of carthamidin and isocarthamidin exhibited 12.5-fold higher DPPH scavenging activity than the substrate—naringenin.

## Conclusions

On the basis of our results, we can conclude that biotransformation potential of *Rhodotorula marina* can be used in a new industrial approach for improvement of flavanone antioxidant properties. We are currently working on the use of cheap components of the cultivation medium for biotransformation involving *R. marina*.
